# *CGRP* neuropeptide levels in patients with endometriosis-related pain treated with dienogest: a comparative study

**DOI:** 10.1186/s12905-024-03095-y

**Published:** 2024-04-24

**Authors:** Shahla Chaichian, Ziba Dehghan Firoozabadi, Samaneh Rokhgireh, Kobra Tahermanesh, Abolfazl Mehdizadeh Kashi, Azam Govahi, Sara Minaeian, Mehdi Mehdizadeh, Marziyeh Ajdary

**Affiliations:** 1 Endometriosis Research Center, Iran University of Medical Sciences, Tehran, Iran; 2 Iranian Scientific Society of Minimally Invasive Gynecology, Tehran, Iran; 3 Antimicrobial Resistance Research Center, Institute of Immunology and Infectious Diseases, Iran University of Medical Sciences, Tehran, Iran; 4 Reproductive Sciences and Technology Research Center, Department of Anatomy, Iran University of Medical Sciences, Tehran, Iran

**Keywords:** Dienogest, *CGRP*, Pain, Endometriosis

## Abstract

**Background and objective:**

Endometriosis (EM) involves the peripheral nervous system and causes chronic pain. Sensory nerves innervating endometriotic lesions contribute to chronic pain and influence the growth phenotype by releasing neurotrophic factors and interacting with nearby immune cells. Calcitonin gene-related peptide (*CGRP*), a pain-signaling neurotransmitter, has a significant role. This study examines the effect of Dienogest (DNG), a hormone therapy used for managing EM -related pain, on serum *CGRP* levels in EM patients.

**Materials and methods:**

The Visual Analog Scale (VAS) assessed pain in diagnosed EM. individuals. Serum samples were obtained to measure *CGRP* concentration. Participants received a 2 mg/day oral dose of DNG for six months as prescribed treatment. Additional serum samples were collected after this period to measure *CGRP* levels.

**Results:**

In the EM group, 6.7%, 33.3%, and 20% had ovarian EM, ovarian plus uterosacral, and ovarian plus bladder, respectively. The EM group showed higher *CGRP* serum levels than the control group (80.53 ± 16.13 vs. 58.55 ± 6.93, *P* < 0.0001). Still, after drug administration, *CGRP* serum levels significantly decreased compared to pre-treatment levels (69.66 ± 11.53 vs. 80.53 ± 16.13, *P* < 0.05). The EM group showed higher pain compared to the control group (7.93 ± 1.58 vs. 0.13 ± 0.35, *P* < 0.0001), but after drug administration, pain significantly decreased compared to pre-treatment levels (1.00 ± 2.00 vs. 7.93 ± 1.58, *P* < 0.05).

**Conclusion:**

DNG administration reduces pain and serum *CGRP* levels in EM patients, offering the potential for innovative treatments and tailored options. Understanding neurotransmitter roles and drug effects can aid in discovering more effective modulators for these pathways.

## Introduction

EM is a prevalent condition characterized by ectopic endometrial tissue outside the endometrium and myometrium. It affects approximately 10% of women of reproductive age, with a higher prevalence observed among women aged 20–50 experiencing chronic pelvic pain and infertility [1]. Despite its prevalence, the precise pathogenesis of EM remains unclear, and optimal treatment strategies are yet to be determined. Various pathogenic theories have been proposed, including retrograde menstruation, coelomic metaplasia, Müllerian remnants, and endometrial stem cell implantation. However, none fully explain the diverse phenotypes and disease severity observed in EM [2]. It is likely that a combination of factors, including genetic and environmental influences, along with menstrual flow, contribute to disease development and phenotypic expression [3, 4].

EM often manifests with severe pain attacks, suggesting disruption of inflammatory signaling pathways and neurotransmitter release, particularly those involved in pain modulation, such as *CGRP*. Dysmenorrhea, characterized by cyclic pain occurring before and during menstruation, is a common symptom associated with EM. Hormonal fluctuations throughout the menstrual cycle are believed to contribute to these pain attacks. Although the relationship between estrogen levels and *CGRP* remains unclear, studies indicate a potential link between *CGRP* and estrogen in women. *CGRP* is implicated in neurogenic inflammation and has been associated with reduced nitric oxide levels and the exacerbation of pain [5, 6].

Over the past two decades, numerous studies have reported sex differences in pain perception, with women generally experiencing higher pain sensitivity compared to men [7–9]. These differences are thought to be influenced by sex hormones and their effects on pain transmission [10, 11]. *CGRP*, synthesized in both peripheral and central neurons, plays a critical role in regulating vascular dilation and pain signaling [12, 13]. Recent findings have highlighted the selective expression of *CGRP* in spinal afferent axon terminals, underscoring its central role in visceral pain transmission [14]. Several endogenous molecules, such as nerve growth factor *(NGF*), can modulate *CGRP* synthesis and release in damaged nerves or tissues [15, 16]. Furthermore, *CGRP* function can be regulated by estrogenic compounds, as evidenced by the significant decrease in *CGRP* levels among menopausal or older women, which can be restored through hormone therapy [17].

Pain perception originates from primary afferent neurons, and hormonal interventions that suppress ovarian activity and implanted implants are employed as pain management strategies in E.M. Hormonal fluctuations are associated with the release of *CGRP* from the endings of trigeminal afferent neurons. .EM and inflammation [18] cause the release of CGRP from these neurons. The increased density of *CGRP*-positive sensory nerve fibers in damaged tissues indicates the involvement of this neuropeptide in E.M. In addition to promoting the proliferation and growth of EM cells, *CGRP* appears to contribute to neurogenic inflammation in this tissue [18, 19]. Despite these findings, the mechanisms related to *CGRP* have not been investigated in women with EM undergoing hormone therapy. Therefore, our study proposes that DNG, a hormonal analgesic therapy, may alleviate pain by modulating *CGRP* levels in the serum of EM patients. This research aimed to examine the level of *CGRP* as a marker for pain reduction and treatment effectiveness in patients treated with DNG. By doing so, we strive to provide valuable insights for researchers exploring nonhormonal treatment methods, enabling them to develop future drugs with inhibitory properties. *CGRP* has the potential to alleviate pain in patients with E.M. Additionally, we aimed to investigate whether progesterone resistance impacts *CGRP* production.

## Method

### Participants

The study enrolled women between 18 and 45 who met specific inclusion criteria. These criteria required the presence of pain associated with EM, which was confirmed through histological examination using the revised criteria of the American Fertility Society (r-AFS, 1985). The diagnosis of EM in patients was established through diagnostic laparoscopy conducted either three months before the study initiation or therapeutic laparoscopy performed within 12 months before the study initiation. Additionally, participants were required to have a pain score exceeding 5.

Exclusion criteria encompassed several factors. These criteria involved the exclusion of pregnant or lactating individuals, those who had experienced amenorrhea within the three months preceding screening, those requiring primary surgical treatment for EM, and those who had previously received hormonal agents such as GnRH agonists for a minimum of six months or had not used progestin, danazol (for at least three months), or oral contraceptives (one month before screening). Furthermore, patients with abnormal gynecological tissue findings, abnormal pap smears within the last three months, a family history of osteoporosis, or a history of anticonvulsant or corticosteroid use were excluded from the study.

For the control group, 15 healthy fertile women were selected based on sonography findings demonstrating the absence of EM symptoms in the pelvic region.

### Study design

**Fig. 1:**
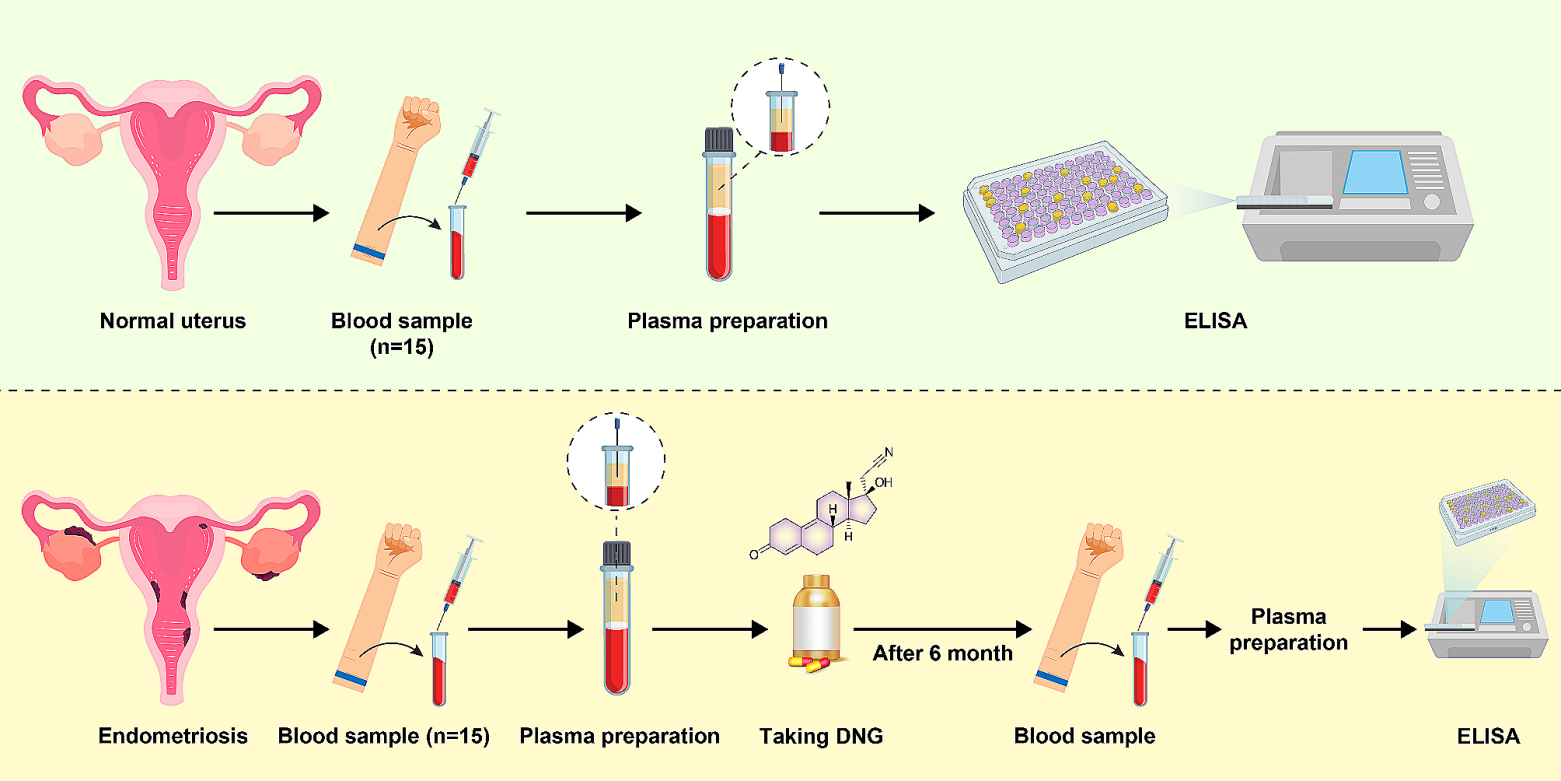
Illustration depicting the schematic representation of patient categorization in the study

The study was designed as a 6-month cohort study to compare the effects of DNG with a control group. Patients were administered oral dienogest at a daily dose of 2 mg, consistently simultaneously each day. The first dienogest pill was taken on the initial day of menstrual bleeding (Fig. 1). The study was conducted at Hazrat Rasool Akram (PBUH) Hospital from June 2022 to August 2023.

### VAS score evaluation

Pelvic pain was evaluated using a visual analog scale (VAS) ranging from 0 mm (indicating no pain) to 100 mm (indicating excruciating pain) [20]. This assessment measured the change in hip pain levels before and six months after the administration of DNG. Furthermore, any self-reported adverse events during the treatment period were meticulously documented.

### Blood sample collection

Serum samples were obtained from all patients before and six months after the initiation of drug administration. Samples were collected during the proliferative phase for both the case and control groups. To measure the serum levels of *CGRP*, peripheral blood samples were initially drawn into sterile tubes containing a coagulant. Subsequently, the tubes were centrifuged at 3000 g for 10 min to separate the serum. The serum samples were then stored at -80 °C until further analysis.

### Evaluation of CGRP with ELISA

The concentration of *CGRP* was assessed using a commercial enzyme immunoassay kit EISA (Zellbio, Germany) by the manufacturer’s instructions. This two-site immunometric method combining an anti-N-terminal antibody with an anti-C-terminal antibody and exhibiting equal sensitivity toward all human *CGRP* isoforms. The assay has a detection limit of 2.5 pg/ml. To ensure quality control and establish a reference standard, two samples from the initial kit were measured alongside each subsequent kit.

### Statistical analysis

Statistical analysis was performed using a one-way analysis of variance (ANOVA) test and GraphPad Prism software. The data were presented as mean ± standard deviation (SD). A significance level of *P* ≤ 0.05 was considered statistically significant.

## Results

### Study population

**Fig. 2:**
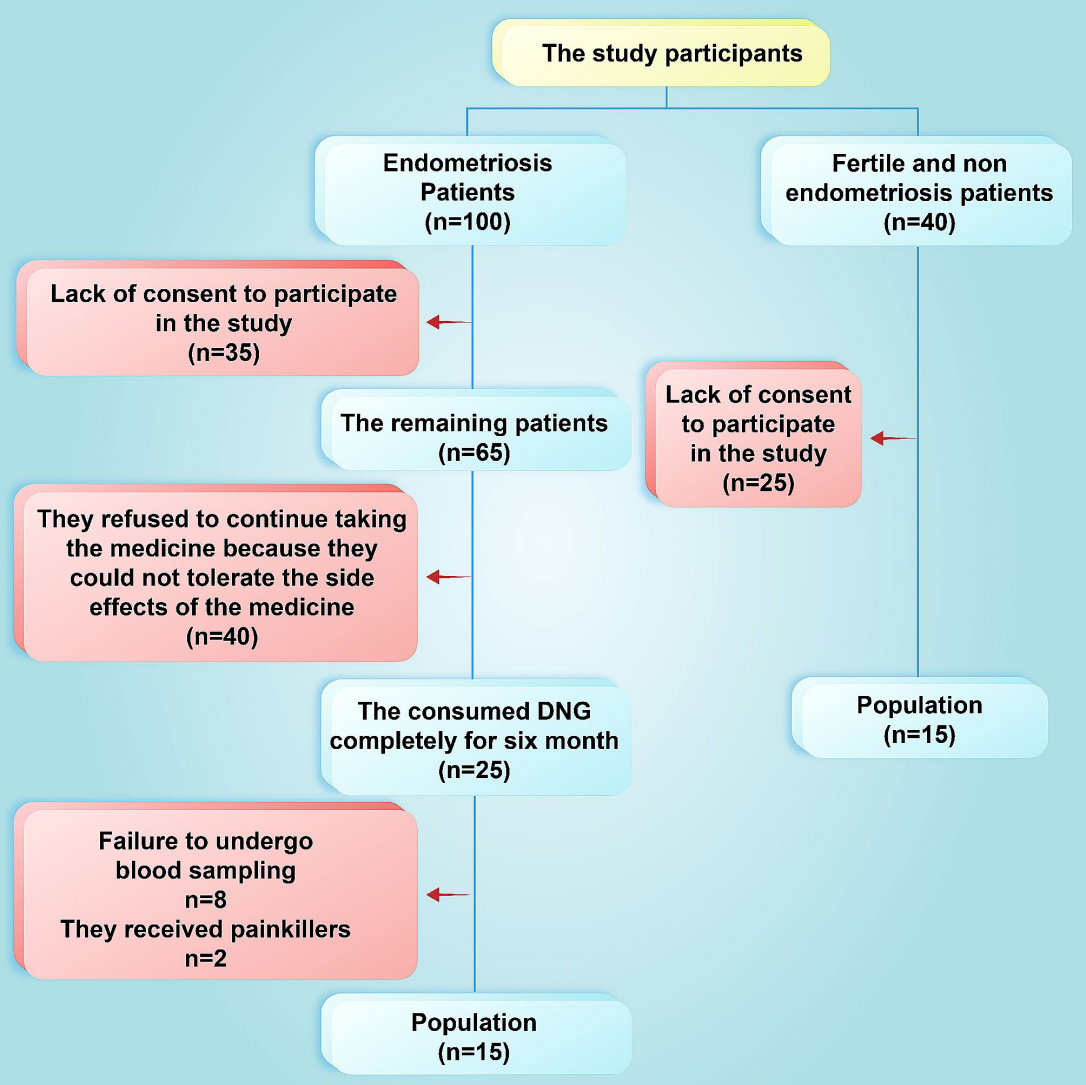
Flowchart illustrating the selection process of EM patients

Out of the initial 100 women who underwent screening. At the onset of the study, 35 participants were excluded, leaving 65 remaining participants. Among these 65 participants, 40 refused to take the medicine because they could not tolerate the side effects of the drug, resulting in a final sample size of 25 participants. Within this group, 8 participants were excluded for not undergoing blood sampling, and 2 participants were excluded for receiving painkillers. Consequently, 15 participants were ultimately included in the data analysis. For the control group, 40 fertile women without EM were initially selected. However, 25 participants discontinued their involvement due to a lack of interest, leaving a final cohort of 15 participants actively participating in the study (Fig. 2).

.M.The EM and control groups were comparable in age, height, weight, and body mass index. Statistical analysis revealed no significant differences between the case and control groups (*p* > 0.05) (Table 1).

**Table 1:** Demographic characteristics of patients

Variables		Endometriosis *group (n* = 15)	Control group (*n* = 15)
**Age (years, mean + SD)**		33.53 ± 3.97	29.73 ± 5.98
***P*** **-value**		0.051	
**Height (cm, mean + SD)**		166.86 ± 7.13	164.00 ± 4.24
***P*** **-value**		0.194	
**Weight (kg, mean + SD)**		68.33 ± 10.13	68.13 ± 5.13
***P*** **-value**		0.94	
**BMI**		24.42 ± 2.59	25.36 ± 2.18
***P*** **-value**		0.293	
**Presence of pain symptoms (n, %)**	pelvic pain	15 (100%)	0 (0%)
	dyspareunia	7 (46.7%)	0 (0%)
	dysmenorrhea	15 (100%)	1(6.6%)
**Classification of endometriosis type (n, %)**	ovarian	7 (46.7%)	0 (0%)
	ovarian plus uterosacral	5 (33.3%)	0 (0%)
	ovarian plus Bladder	3 (20%)	0 (0%)
**adverse events (n, %)**	Depression	7 (53.8%)	0 (0%)
	hair loss	6 (46.2%)	0 (0%)

All patients with EM experienced pelvic pain and dysmenorrhea, with seven individuals (46.7%) also reporting dyspareunia. In the control group, only one person (6.6%) had dysmenorrhea. Among the EM patients, seven individuals (46.7%) had ovarian EM, five individuals (33.3%) had ovarian plus uterosacral EM, and three individuals (20%) had ovarian plus bladder EM. Furthermore, seven individuals (53.8%) reported depression, while six individuals (46.2%) experienced hair loss as a side effect of the medication they were taking (Table 1).

### Changes in serum CGRP levels

**Fig. 3:**
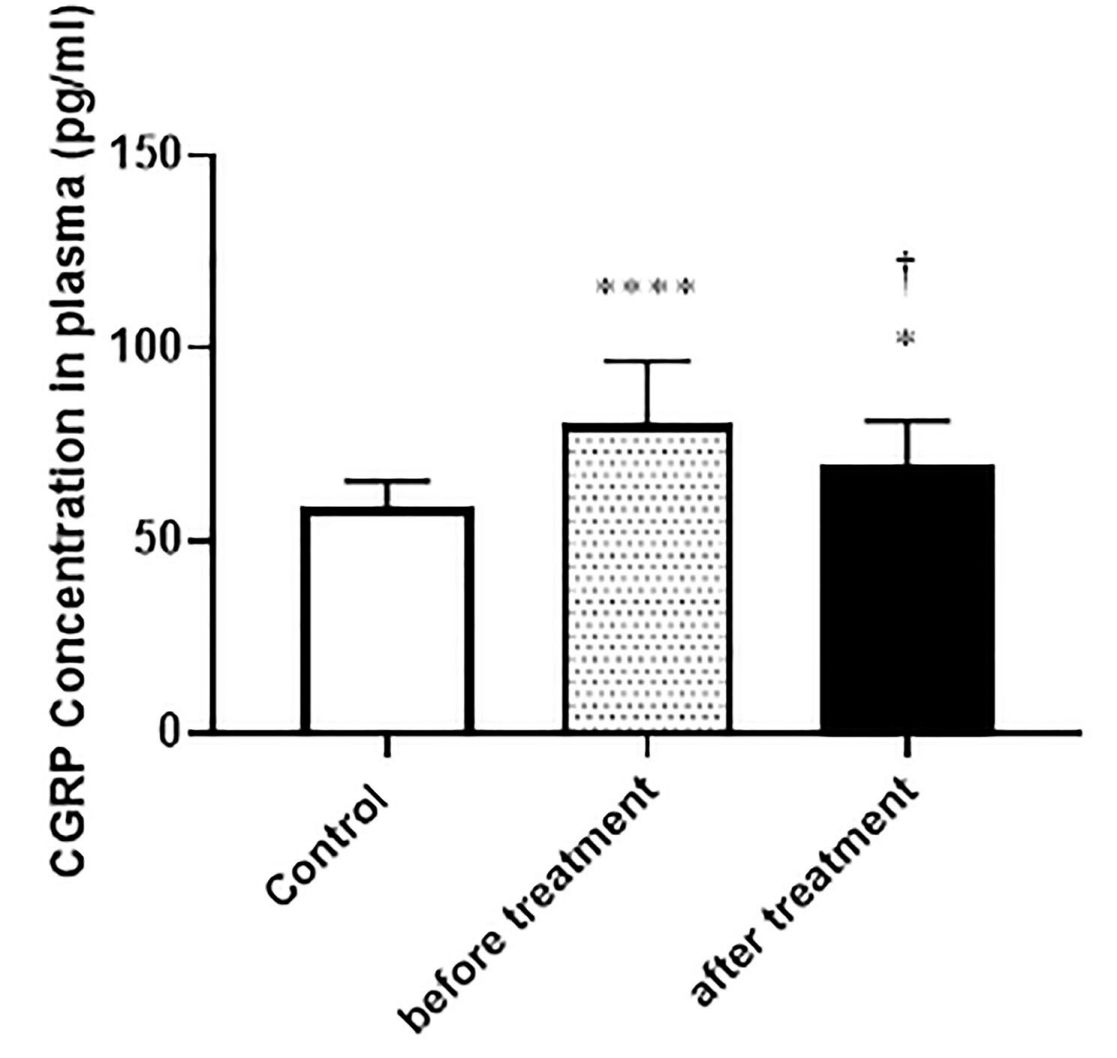
Mean ± SD (*n* = 15 for each group). Comparison of patients’ CGRP serum levels at the study’s beginning and 24 weeks after consuming DNG 2 mg in the treated and control groups. *: comparison with the control group, † denotes comparison between pre and post-treatment. Significance levels: ****P < 0.0001, *P < 0.05, †*P* < 0.05

As depicted in Fig. 3, the average *CGRP* serum levels in the control group were 58.55 ± 6.93. Before receiving DNG treatment, the EM group exhibited a significantly higher average *CGRP* serum level in serum level of 80.53 ± 16.13 compared to the control group (*P* < 0.0001). Following six months of drug administration, there was a significant decrease in the *CGRP* serum levels to 69.66 ± 11.53 compared to pre-treatment levels (*P* < 0.05).

### Patients’ vas scores

**Fig. 4:**
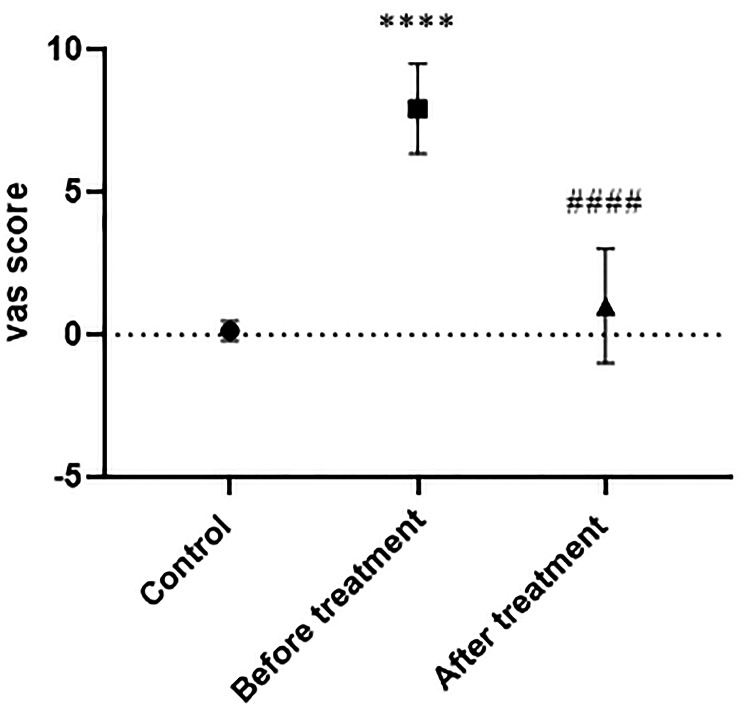
Mean ± SD (*n* = 15 for each group). Comparison of VAS scores at the beginning of the study and 24 weeks after taking DNG 2 mg in the treated and control groups. *: comparison with the control group, and # denotes comparison between pre and post-treatment. Significance levels: **** *P* < 0.0001, #### *p* < 0.0001

As shown in Fig. 4, assessment of pelvic pain, dysmenorrhea pain, and dyspareunia pain using the VAS scale revealed an average score of 0.13 ± 0.35 in the control group. Before receiving DNG treatment, the EM group displayed a significantly higher average VAS score of 7.93 ± 1.58 compared to the control group (*P* < 0.0001). Following six months of drug administration, the VAS score significantly decreased to 1.00 ± 2.00 compared to pre-treatment levels (*P* < 0.0001).

## Discussion

The present study demonstrates that the levels of *CGRP* are influenced by the administration of DNG in patients with E.M. Women with EM exhibit higher serum levels of *CGRP* compared to healthy individuals. Changes influence the fluctuations in CGRP levels in female hormones, and this study sheds light on the impact of hormone therapy on pain levels in EM patients. Several factors contribute to regulating *CGRP* homeostasis, including ovarian sex hormones. Previous research by Stevenson et al. 1986 indicated that *CGRP* levels increase during pregnancy and return to normal levels after delivery [21].

Furthermore, studies by Adewuyi et al. have highlighted the involvement of mitogen-activated protein kinase (*MAPK*) and *TNF-α* signaling pathways in *CGRP* modulation [22]. Inflammation triggers the activation of *MAPK*, which subsequently leads to the release of *CGRP* from nerve endings. *CGRP* plays a significant role in neuropathic pain as it promotes the release and distribution of inflammatory factors from meningeal mast cells [23, 24]. Moreover, *CGRP* induces the release of inflammatory mediators such as bradykinin and prostaglandins from nerve endings and immune cells [25–27]. The release of *CGRP* also contributes to increased blood flow in trigeminal tissues. These findings suggest that blocking the pathophysiological activities of *CGRP* through *CGRP* receptor antagonists may have implications for the treatment of neuropathic pain [28].

**Fig. 5:**
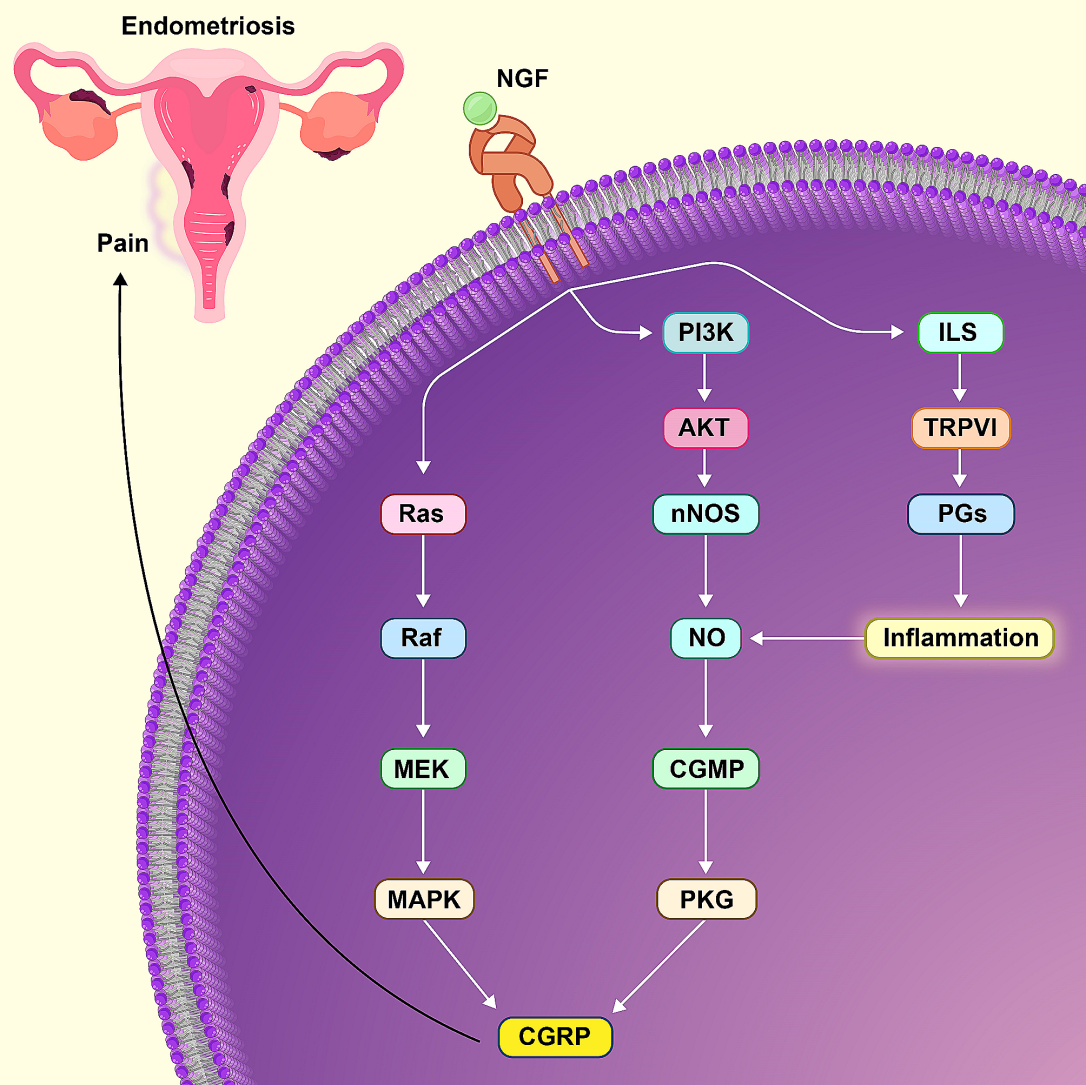
*CGRP* marker release signaling pathway

.M.This study observed that the serum levels of CGRP were higher in women with EM than in healthy individuals. Previous studies have also demonstrated that *CGRP* is secreted from EM lesions, leading to the release of Ca^+ 2^ and increased inflammation [29, 30] (Fig. 5).

Other studies have shown that one of the neuropeptides released by EM is NPSR1, which increases pain through the stimulation of chemokines-cytokine pathways in these patients [31]. In this study, it is shown in Fig. 5 that *CGRP* increases due to interleukins and increasing NO. Marc Passover, et al. in 2009 stated that neuropeptide-Y increases pain in EM patients through the MEK pathway [32]. In this study, it was shown in Fig. 5 that MAPKs cause the release of *CGRP*. The showed that neuropeptides that are elevated through EM pain have a pathway similarM to the *CGRP* pathway.

In a study conducted by Bianca Raffaelli et al. in 2021, the role of *CGRP* in women with both EM and migraine was investigated. Blood serum samples were collected during the menstrual cycle and on the fifteenth day of ovulation. The results revealed significant differences in plasma *CGRP* levels between the menstruation and ovulation periods among the groups (*p* = 0.007). Women with both migraine and EM exhibited an increase in *CGRP* during the menstrual phase compared to the ovulation phase, whereas the healthy control group displayed lower *CGRP* levels. Furthermore, the amount of *CGRP* differed before ovulation across the various groups, with higher levels observed in the control group compared to the other groups [33].

In a study by Vincenzo Pota et al. 2016, the involvement of estrogen hormones in visceral pain transmission and the regulation of *CGRP* levels were investigated. The findings suggest that the release of *CGRP*, regulated by 17β-estradiol, can partially explain the gender difference in sensitivity to visceral pain. Moreover, it was found that women experience higher levels of pain [34].

These studies contribute to identifying pain pathways and significant markers within these pathways. The authors of this article anticipate identifying miRNAs involved in reducing *CGRP* serum levels and alleviating pain in the future. These results could introduce therapeutic approaches for reducing EM-related pain in the medical industry and the wider community.

However, one of the limitations of this study is the small sample size. Still, because the samples were collected from a reference hospital, patients came from different cities in Iran, and visiting once every three months was problematic for the patients. For a better comparison, blood sampling should be done every three months to measure the CGRP level and the pain amount. It is suggested that researchers pay attention to these points in a similar study.

## Conclusion

The administration of DNG effectively reduces pain and *CGRP* serum levels in patients with E.M. A comprehensive understanding of the role of neurotransmitters and the impact of drugs targeting these pathways can pave the way for novel treatment approaches and more suitable therapeutic options. This knowledge may lead to developing new medicines that exhibit enhanced efficacy in modulating these pathways, thereby improving the treatment outcomes for individuals with E.M.

## Data Availability

The underlying data supporting the results of our study can be found in the manuscript.
